# Barriers, Resources and Reserves Shaping Older Migrants’ Access to Healthcare: A Research Imperative for Switzerland

**DOI:** 10.3389/ijph.2025.1608753

**Published:** 2025-09-01

**Authors:** Paolo Martinelli

**Affiliations:** ^1^ Institute of Psychology, University of Lausanne, Lausanne, Switzerland; ^2^ School of Engineering and Management Vaud, HES-SO, Yverdon-les-Bains, Switzerland; ^3^ Swiss Centre of Expertise in Life Course Research, LIVES Centre, Lausanne, Switzerland

**Keywords:** older migrants, healthcare access, health inequalites, resources and reserves, Switzerland

**The IJPH series “Young Researcher Editorial” is a training project of the Swiss School of Public Health.**

Migrants are highly exposed to unmet healthcare needs in Europe, with rates varying between countries [1]. In Switzerland, almost 10% of non-European migrants experience unmet healthcare needs, rising to nearly 20% for specific healthcare services such as dental care [1]. Older migrants are particularly vulnerable to these hardships [1]. Their life course and migration pathways have led to an accumulation of adverse social determinants of health, resulting in inequalities of both health status and access to the receiving society’s healthcare system.

Older migrants encounter barriers in accessing healthcare, in the medical treatment they receive, and in the reimbursement of health services. Compared to older native-born individuals, older migrants are less likely to have a family doctor and, when they do, are less inclined to consult for illness [2]. Ailing older migrants also receive fewer prescriptions than native older citizens [3]. When they access formal healthcare, cultural and systemic barriers can further prevent them from obtaining appropriate support. For example, scholars found that healthcare professionals were less likely to monitor and identify carcinomas and adenomas in older patients with a migratory background [4]. Moreover, older migrants may be less aware of medical reimbursement and more exposed to insurance precariousness, leading them to avoid specific treatments such as dental care [5]. These adversities do not affect all older migrants in the same way: those from non-European countries, with low socio-economic status and limited social support networks are particularly exposed to unmet healthcare needs.

Various national and international programs have been launched to reduce these inequalities and enable migrants to have fair and appropriate access to healthcare. However, these programs mainly target refugees and undocumented migrants, and largely overlook the challenges encountered by older migrants. For instance, in Switzerland, the *Swiss Hospitals for Equity* and Migrant Care Unit (i.e., *Unité de Soins aux Migrants*–USMi) programs are primarily aimed at migrant groups deemed vulnerable due to their background, young age, and residence status, failing to prioritize older migrants’ healthcare needs.

Without structural support tailored to their needs, older migrants require to mobilize individual, social, and territorial resources and reserves to meet their healthcare needs. Past research has extensively highlighted the role of language skills and health literacy among older migrants, enabling them–for instance–to engage in more frequent telemedicine consultations [6]. Yet, individual resources and reserves are linked to their health-related social capital. Older migrants may benefit from what scholars have defined as ‘healthcare convoys’: heterogeneous social networks that help them meet their health needs [7]. Combining family members, friends, and health professionals from the receiving society or country of origin, these networks provide older migrants with appropriate health information, transportation assistance, language and interpretation support in healthcare interactions [7]. Older migrants may also benefit from territorial resources and reserves: limited social and geographical spaces fostering various forms of health support, such as third places (e.g., cafés, community organizations), transitory zones (e.g., sidewalks, bus stops), and semi-public spaces (e.g., balconies, backyards) [8]. These spaces bring together non-family networks including neighbors, volunteers, and members of the religious community which provide older adults with various acts of informal care [8]. Such forms of care could enable older migrants to receive daily assistance (e.g., grocery shopping), fulfill their basic needs (e.g., offered meals), reduce their social isolation, and express their healthcare concerns.

These individual, social, and territorial resources and reserves contribute simultaneously to improve older migrants’ care-seeking strategies and the functioning of the healthcare system, such as the overuse of emergency care instead of primary care [9]. Accordingly, such resources and reserves could have positive spin-offs, including the mitigation of health inequalities and of the receiving society’s public healthcare expenditure [10].

Migrants increasingly tend to settle in the receiving society as they age, and late-life migration is sharply growing. Yet, only scant research has examined how older migrants facing healthcare barriers meet their health needs. It is therefore of utmost importance for future research to understand the dynamics and interconnectedness of individual, social, and territorial resources and reserves enabling older migrants to access healthcare in spite of the barriers they encounter. To this end, with one in four older citizens being born outside the country and a highly fragmented and complex-to-navigate healthcare system, Switzerland provides a fertile ground for research. Studying this specific setting could show how older migrants manage their healthcare needs in a high-demanding system, and provide beneficial insights for other countries facing similar demographic and structural challenges, such as Germany and the Netherlands. In Switzerland, however, no study has yet addressed the formal and informal healthcare barriers, resources, and reserves experienced and mobilized by older migrants. Such efforts are a prerequisite for the development of comprehensive, multilevel support–ranging from transcultural training for healthcare professionals to the implementation of targeted programs that promote equitable access to healthcare for this population.

## Data Availability

The original contributions presented in the study are included in the article/supplementary material, further inquiries can be directed to the corresponding author.

## References

[1] Eurostat. Migrants Integration Statistics – Health (2025). Available online at: https://ec.europa.eu/eurostat/statistics-explained/index.php?title=Migrant_integration_statistics_-_health#Highlights (Accessed May 12, 2025).

[2] Gimeno-Feliu LA Macipe-Costa RM Dolsac I Magallón-Botaya R Luzón L Pardos-Torres A Frequency of Attending Primary Care Clinics by the Immigrant versus Autochthonous Population. Atencion Primaria (2011) 43(10):544–50. 10.1016/j.aprim.2010.09.014 21536353 PMC7025054

[3] Franchi C Baviera M Sequi M Cortesi L Tettamanti M Roncaglioni MC Comparison of Health Care Resource Utilization by Immigrants versus Native Elderly People. J Immigrant Minor Health (2016) 18(1):1–7. 10.1007/s10903-014-0152-2 25576178

[4] Turrin A Zorzi M Giorgi Rossi P Senore C Campari C Fedato C Colorectal Cancer Screening of Immigrants to Italy. Figures from the 2013 National Survey. Prev Med (2015) 81:132–7. 10.1016/j.ypmed.2015.08.016 26358527

[5] Reyes AM Hardy M. Health Insurance Instability Among Older Immigrants: Region of Origin Disparities in Coverage. The Journals Gerontol Ser B (2015) 70(4):303–13. 10.1093/geronb/gbu218 25637934 PMC4415080

[6] Rodríguez-Fernández JM Hoertel N Saner H Raji M. Acculturation and Disparities in Telemedicine Readiness: A National Study. Int J Aging Hum Dev (2024) 99(1):96–114. 10.1177/00914150231219259 38111265 PMC11295414

[7] Kemp CL Ball MM Perkins MM. Convoys of Care: Theorizing Intersections of Formal and Informal Care. J Aging Stud (2012) 27(1):15–29. 10.1016/j.jaging.2012.10.002 23273553 PMC3611594

[8] Gabauer A Glaser M Christensen L Lehner JM Jing J Lundberg S. Geographies of Aging: Hidden Dimensions of Care in Stockholm, Vienna, and Zurich. In: Gabauer A Knierbein S Cohen N Lebuhn H Trogal K Viderman T, editors. Care and the City: Encounters with Urban Studies. Routledge (2021). p. 171–82.

[9] Acquadaro-Pacera G Valente M Facci G Kiros BM Della Corte F Barone-Adesi F Exploring Differences in the Utilization of the Emergency Department between Migrant and Non-Migrant Populations: A Systematic Review. BMC Public Health (2024) 24:963. 10.1186/s12889-024-18472-3 38580984 PMC10996100

[10] Loi S Li P Myrskylä M. Unequal Weathering: How Immigrants’ Health Advantage Vanishes over the Life-Course. J Migration Health (2025) 11:100303. 10.1016/j.jmh.2025.100303 39911450 PMC11795556

